# Usability and Accuracy of a Smartwatch for the Assessment of Physical Activity in the Elderly Population: Observational Study

**DOI:** 10.2196/20966

**Published:** 2021-05-05

**Authors:** Matteo Martinato, Giulia Lorenzoni, Tommaso Zanchi, Alessia Bergamin, Alessia Buratin, Danila Azzolina, Dario Gregori

**Affiliations:** 1 Unit of Biostatistics, Epidemiology and Public Health Department of Cardiac-Thoracic-Vascular Sciences and Public Health University of Padova Padova Italy; 2 Department of Biology University of Padova Padova Italy; 3 Department of Molecular Medicine University of Padova Padova Italy; 4 Department of Translational Medicine University of Eastern Piedmont Novara Italy

**Keywords:** wearable devices, elderly, physical activity, smartwatches

## Abstract

**Background:**

Regular physical activity (PA) contributes to the primary and secondary prevention of several chronic diseases and reduces the risk of premature death. Physical inactivity is a modifiable risk factor for cardiovascular disease and a variety of chronic disorders such as diabetes, obesity, hypertension, bone and joint diseases (eg, osteoporosis and osteoarthritis), depression, and colon and breast cancer. Population aging and the related increase in chronic diseases have a major impact on the health care systems of most Western countries and will produce an even more significant effect in the future. Monitoring PA is a valuable method of determining whether people are performing enough PA so as to prevent chronic diseases or are showing early symptoms of those diseases.

**Objective:**

The aim of this study was to estimate the accuracy of wearable devices in quantifying the PA of elderly people in a real-life setting.

**Methods:**

Participants aged 70 to 90 years with the ability to walk safely without any walking aid for at least 300 meters, who had no walking disabilities or episodes of falling while walking in the last 12 months, were asked to walk 150 meters at their preferred pace wearing a vívoactive HR device (Garmin Ltd) and actual steps were monitored and tallied by a researcher using a hand-tally counter to assess the performance of the device at a natural speed. A Bland-Altman plot was used to analyze the difference between manually counted steps and wearable device–measured steps. The intraclass correlation coefficient (ICC) was computed (with a 95% confidence interval) between step measurements. The generalized linear mixed-model (GLMM) ICCs were estimated, providing a random effect term (random intercept) for the individual measurements (gold standard and device). Both adjusted and conditional ICCs were computed for the GLMM models considering separately the effect of age, sex, BMI, and obesity. Analyses were performed using R software (R Foundation for Statistical Computing) with the rms package.

**Results:**

A total of 23 females and 26 males were enrolled in the study. The median age of the participants was 75 years. The Bland-Altman plot revealed that, excluding one observation, all differences across measurements were in the confidence bounds, demonstrating the substantial agreement between the step count measurements. The results were confirmed by an ICC equal to .98 (.96-.99), demonstrating excellent agreement between the two sets of measurements.

**Conclusions:**

The level of accuracy of wearable devices in quantifying the PA of elderly people in a real-life setting that was found in this study supports the idea of considering wrist-wearable nonmedical devices (widely available in nonspecialized stores) as reliable tools. Both health care professionals and informal caregivers could monitor the level of PA of their patients.

## Introduction

There is remarkable evidence that regular physical activity (PA) contributes to the primary and secondary prevention of several chronic diseases and reduces the risk of premature death [1]. There is a linear relation between the amount of PA and health benefits, such that the most physically active people are at the lowest risk [1]. However, the greatest improvements in health status are recorded when people who are the least fit become physically active [1].

Physical inactivity is a modifiable risk factor for cardiovascular disease and a variety of chronic disorders such as diabetes, obesity, hypertension, bone and joint diseases (eg, osteoporosis and osteoarthritis), depression, and colon and breast cancer [2]. In this framework, primary prevention plays a key role in the management of these diseases. Moreover, health promotion programs should target people of all ages, since the risk of developing chronic diseases starts in childhood and increases with age [1].

Population aging, and the related increase in chronic diseases, has a major impact on the health care systems of most Western countries and will produce an even more significant effect in the future. It has been calculated that, in 2001, chronic diseases accounted for approximately 60% of the 56.5 million total reported deaths in the world and for approximately 46% of the global burden of disease [3]. Almost half of chronic disease-related deaths are attributable to cardiovascular diseases. Obesity and diabetes are also showing worrying trends, not only because they already affect a large part of the population but because they have started to appear earlier in people’s lives. Chronic diseases can be prevented with a healthy diet, avoidance of tobacco products, and regular PA [4]. Moreover, chronic diseases lead to a limitation of mobility and PA of affected persons, with a slow, progressive, and sometimes unnoticed entry mechanism, culminating in a reduction of autonomy. Therefore, monitoring PA is a valuable parameter for determining whether people are performing enough PA to prevent chronic diseases or if they are showing early symptoms of those diseases [5].

In recent years, several methods of monitoring PA and sedentary behavior have been proposed. Self-reporting is a simple tool for assessing PA via the completion of questionnaires, interviews, and surveys [6]. Alternatively, PA can be monitored by diaries or logs, where information on all forms of activity are recorded day by day. Those tools require a detailed description of the performed activity, including its intensity and duration. This method could produce useful health-related data, but the approach requires considerable worktime to produce standardized data. The recording of relevant data should be relatively simple and cover several days to avoid any potential bias [7].

Videorecording, adopting static cameras, wearable cameras, or low-cost motion-sensing input systems such as Kinect (Microsoft Corp), is another example of an autonomous data collection method [8]. Although this approach has a definite role in the assessment of activity patterns with the advantage of direct observation, it is unlikely to be practicable for large groups of individuals, requiring a great amount of resources to analyze and quantify videorecordings.

Heart rate monitors are low-cost tools, and heart rate can be used as a good quality proxy for PA, but it is not a precise indicator of energy expenditure unless proper individual calibration is performed. Gold standard techniques for measuring energy expenditure are based on the double-labeled water method or indirect calorimetry measuring oxygen uptake, carbon dioxide production, and cardiopulmonary parameters, but these techniques, although accurate, require specialized training and are expensive and not suitable for large-scale studies.

Advances in technology are facilitating researchers to quantify PA, and accelerometry-based activity monitors may be more suitable methods. Accelerometers are small and easy-to-use devices that track movement in 1 to 3 dimensions (ie, anteroposterior, vertical, and mediolateral). Using these tools, people can measure the frequency, intensity, and duration of PA. They are comfortable to wear, relatively inexpensive, and accurate compared with research-grade PA devices [9]. Accelerometers are technically more advanced than pedometers, and being multiaxial, they can measure horizontal, lateral, and vertical movements. These devices can be used to measure steps, activity counts, energy expenditure, posture, walking, and different intensities of movement. In addition, the reliability and validity of accelerometer data are generally high [10].

Recently, more attention has been paid to wrist-worn accelerometers. They are convenient and comfortable to wear, and patient compliance improves significantly while participating in studies requiring prolonged measurements. At the same time, these systems provide a high level of accuracy. Furthermore, the integration of additional motion sensors can be considered to increase the overall performance. Such integrated sensors may include gyroscopes, magnetometers, barometers, GPS devices, and physiological sensors (eg, heart rate) devised to improve the assessment and detection of specific indicators.

In this sense, wearable motion detectors might be the most promising technology for enabling an automatic, continuous, and long-term assessment of subjects in free-living environments. In addition, the obtained PA parameters can be shared with health care providers and insurance platforms to better describe behavioral patterns and functional ability in high-risk subjects, thus providing important feedback regarding the overall health status of an individual and even the prediction of potential adverse health events.

Nevertheless, few data are available on the accuracy and reliability of such devices, which are often built for active sports or athletes, in an elderly population. Some authors have validated this device on healthy older adults in studies including 20 participants [11].

In previous studies, wrist-worn devices showed poorer agreement to reference devices, suggesting that researchers should consider that not all consumer-level activity monitors are equal in terms of accuracy, design, and function [12].

Current public health recommendations for adults aged 65 years and older in general good health (moderate or vigorous intensity activity) could be seen challenging for many older people, and the health benefits of light intensity activities have not been defined even if light activities, including walking, account for the biggest part of daily activity in older populations [13].

Moreover, results of previous studies have been obtained in experimental settings and/or comparing device performance with data provided by reference instruments and in younger populations; they should be confirmed in other settings and with other devices [14] in older subjects to be considered for clinical application in the elderly.

PA research performed with validated but commercially available smartwatches holds the potential to address this gap with a more comprehensive assessment of the benefit of the overall amount of time spent ambulatory daily, and thus help shape future interventions specifically designed for increasing daily PA in older adults [13].

The study aimed to assess the feasibility of extending the use of these devices for monitoring the health of the elderly population and estimating the accuracy of wearable devices in quantifying the PA of elderly people on a larger sample of 49 subjects in a setting similar to a real-life condition excluding only individuals with a diagnosis of atrial fibrillation and current anticoagulant treatment.

## Methods

### Study Design

In this study, a commercially available smartwatch has been compared with direct observation of step counts, a metric successfully used in interventions to improve clinical outcomes [15] in a real-life, noninterventional, controlled setting (a walk at own pace in a daily attended location).

Aiming to perform a study that could be considered a starting point to extend the use of these devices for monitoring the health of the elderly population, a more protected experimental setting was chosen; however, the study setting was not fully controlled but resembled a real-life context: the proposal for participation in the study and the execution were immediate and conducted in a local market, a setting frequented by the participants. Moreover, the subjects freely walked at their own pace.

The study was conducted in flat areas previously marked with fixed distances on the ground using a Mini Measuring Wheel odometer (Group Silverline Ltd) during daily life circumstances in different cities in northeastern Italy. The path was linear and previously measured.

Participants met the following criteria: signed the informed consent form, aged between 70 and 90 years, able to walk safely and without any walking aid for at least 300 meters, no history of episodes of falling while walking in the last 12 months, no current diagnosis of atrial fibrillation, and no current anticoagulant treatment. Enrollment and the assessment were performed on the same day.

Participating subjects were asked to walk 150 meters at their preferred pace to assess the performance of the device at a natural speed [16]. Subject characteristics such as sex, age, weight, height, health conditions, and the number of steps on the path were collected.

Actual steps were monitored and tallied by a researcher using a hand-tally counter. The hand-tally count has been used as a criterion measure for manually measuring steps [17].

### Study Device

In this study, a vívoactive HR smartwatch (Garmin Ltd) was programmed with participant sex, age, weight, and height and fitted on the left wrist according to the user manual. The device was designed for monitoring physical activity, especially outdoors, and has been validated in a real-life setting in other studies on the adult population [18]. The device tested in this study can also provide raw data regarding heart rate, number of steps, sleep quality, and activity (walking) session duration. In the literature, it has been demonstrated that the vívoactive HR was more accurate at reflecting step count across a broader range of walking cadences than other devices [19] also considering different age groups and during various walking conditions, even during slow walking [20].

The vívoactive HR is one of the few devices compliant with the new technological standards for physical activity monitors [21]. This device allows a detailed download of the raw data relating to each walking or training session, which allows accurate tracking of the physical activity of the elderly subject for health monitoring purposes. The activity monitor was started simultaneously with the start of the test. This time point was also recorded on the case report form by a different researcher to allow identification of the start point of the measurement [22]. After testing was completed, data were downloaded onto a personal computer via USB drive for postprocessing and analysis.

### Sample Size

The sample size computation was performed using the method proposed by Bonett [23] for estimating intraclass correlation coefficients (ICCs). Two different step counting methods (a gold standard manual evaluation and wearable device counting) were considered to tailor the study design [24]. An ICC value of .80 was used as the expected agreement for the sample size computation, as indicated in the literature for other studies evaluating the agreement between device step counting and the manual counting gold standard [25]. A sample size of 49 subjects was considered sufficient to estimate the expected ICC with a precision of 0.10 based on a 95% confidence interval.

### Statistical Analysis

Descriptive statistics were reported as medians and interquartile ranges for continuous variables and counts and percentages for categorical variables. The Wilcoxon Kruskal-Wallis test was performed for continuous variables, and the Pearson chi-square test was performed for categorical variables.

A Bland-Altman plot was used to analyze the difference between manually counted steps and wearable device–measured steps. The ICC was also computed (with a 95% confidence interval) between the step measurements.

Generalized linear mixed-model (GLMM) ICCs were also estimated, providing a random effect term (random intercept) for the individual measurements (gold standard and device). Both adjusted and conditional ICCs [26] were computed for the GLMM models considering separately the effects of age, sex, BMI, obesity (BMI >30), and session duration in minutes.

The adjusted ICC only considers the random effects in the computation, while the conditional ICC also takes the fixed effects variances into account and evaluates how much the covariate variable explains the portion of the variability in the grouping structure (random intercept).

The likelihood ratio test (LRT) was performed comparing the goodness of fit of the separate covariate-adjusted models with that of the intercept model (null model).

The covariates indicating significant goodness-of-fit improvement (ie, a significant covariate effect on the agreement between measures) in comparison with the null model were selected to perform a generalized linear model (GLM) on the number of misclassified steps (ie, the absolute value of the difference between the gold standard and device measurement). The negative binomial parametrization was considered to adjust the model estimates for overdispersion. The dispersion test was also performed as indicated in the literature [27]. The model fit was evaluated by reporting the residual Q-Q plots. Analyses were performed using R (R Foundation for Statistical Computing) [28] with the rms package [29].

## Results

A total of 23 females and 26 males were enrolled in the study. The median age of the participants was 75 years (Table 1). A considerable proportion of participants included in the sample were obese (33/49, 67%), and the majority (20/33, 61%) were male. The step counts for both measurement criteria were greater for female participants than for male. Many older people could not participate due to walking problems or because they used walking aids. Many people approached said they did not have time to listen to information on the study, and this affected recruitment. The relatively younger subjects were found to be more willing to receive information on the study than older people. On the other hand, study participation was very high: in 50 people selected after checking the inclusion and exclusion criteria, only one refused to participate in the study.

**Table 1:** Descriptive table of patient characteristics and step counts according to sex^a^.

Characteristic			Females (n=23)		Males (n=26)		Combined (n=49)		*P* value
**Patient demographic data**									
	Age in years, median (IQR^b^)	75.00 (73.00-79.00)		72.00 (71.00-76.75)		74.00 (71.00-77.00)		.16	
	Weight (kg), median (IQR)	68.00 (62.50-76.50)		81.00 (75.00-90.00)		75.00 (68.00-85.00)		<.001	
	Height (m), median (IQR)	1.62 (1.58-1.66)		1.72 (1.70-1.77)		1.69 (1.62-1.73)		<.001	
	BMI, median (IQR)	25.00 (23.10-28.99)		27.21 (25.51-29.40)		26.99 (24.22-29.36)		.20	
	Obese (BMI >30), n (%)	11 (48)		22 (85)		33 (67)		.006	
**Step counts**									
	Gold standard, median (IQR)	232.0 (214.5-243.5)		211.0 (197.2-221.8)		217.0 (208.0-233.0)		.001	
	Device, median (IQR)	233.0 (216.0-247.0)		212.5 (195.5-221.0)		219.0 (208.0-236.0)		.002	
	Session duration (min), median (IQR)	2.000 (1.900-2.225)		1.950 (1.850-2.062)		1.967 (1.867-2.167)		.16	

^a^The Wilcoxon Kruskal-Wallis test was performed for continuous variables, and the Pearson chi-square test was performed for categorical variables.

^b^IQR: interquartile range.

**Figure 1:**
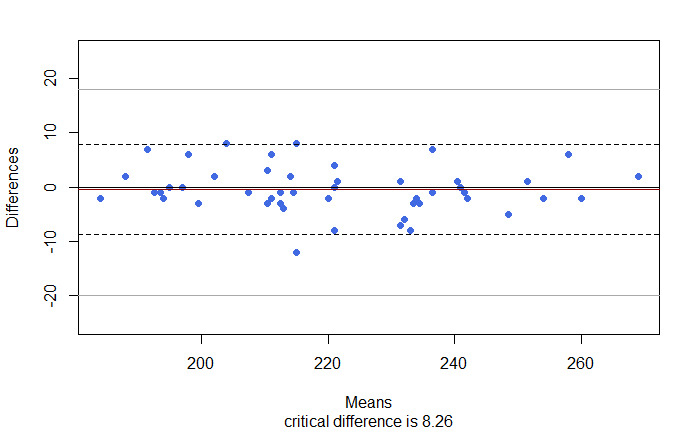
Bland-Altman plot of the difference between the gold standard daily steps and the wearable device measured daily steps (intraclass correlation coefficient .98, 95% CI 0.96-0.99). The red line indicates the mean difference (–0.4). The dotted lines indicate the Bland limit of agreement 1.96=*SD. The dark grey lines indicate a reasonable limit of agreement (-20; 18) as indicated in a study conducted to validate a Fitbit wearable device on an elderly population [31].

The Bland-Altman plot (Figure 1) reveals that, except for one observation, all the differences across measurements were in the confidence bounds, demonstrating substantial agreement between the step count measurements. Other research has been performed to validate wearable devices (Fitbit) on elderly subjects using the manual step count as the gold standard. Following this evidence, a clinically relevant lower limit of the agreement is –20 steps and an upper limit is 18 steps [30]. These limits have been represented in the Bland-Altman plot. All points lie within these boundaries indicating also that the range between the limits of agreement is narrow enough to represent nonclinically significant variation in the outcome. Results were also confirmed by an ICC equal to .98 (.96-.99), demonstrating excellent agreement between the two sets of measurements [25].

The GLMM conditional agreement analysis revealed that the ICC values, after controlling for the predictors, were different after adjusting for age and sex (Table 2). The LRT test revealed that age and sex were the covariates indicating significant improvement in model fit in comparison with the null model.

**Table 2:** Generalized linear mixed-model intraclass correlation coefficients (ICCs) have been estimated, providing a random effect term (random intercept) for individual measurements (gold standard and device). The adjusted ICC considers only the random effects, while the conditional ICC also takes the fixed-effect variances into account when evaluating how much the covariate variable explains the portion of the variation in the grouping structure (random intercept). The *P* value and chi-square test statistics are reported for the likelihood ratio test comparing the goodness of fit of the separate covariate-adjusted models with the intercept-only model (null model).

Model	Adjusted ICC^a^	Conditional ICC	Chi-square LRT^b^	*P* value (LRT)
Null model	.98	—^c^	—	—
Age in years	.98	.77	12	<.001
Sex	.98	.77	11.77	<.001
BMI	.98	.91	3.55	.06
Obesity	.98	.94	1.88	.17
Walking session duration	.98	.98	0.002	.90

^a^ICC: intraclass correlation coefficient.

^b^LRT: likelihood ratio test.

^c^Not applicable.

Considering the aforementioned results, sex and age were included as covariates to model the number of misclassified steps. A GLM with a negative binomial parametrization was considered for the estimation. This approach led to the adjustment of the estimates for the overdispersion component (ϕ). The dispersion test revealed that ϕ was equal to 2.11 and was significantly (*P*<.001) greater than 1 (ϕ = 1 indicates the absence of overdispersion in the data).

**Figure 2:**
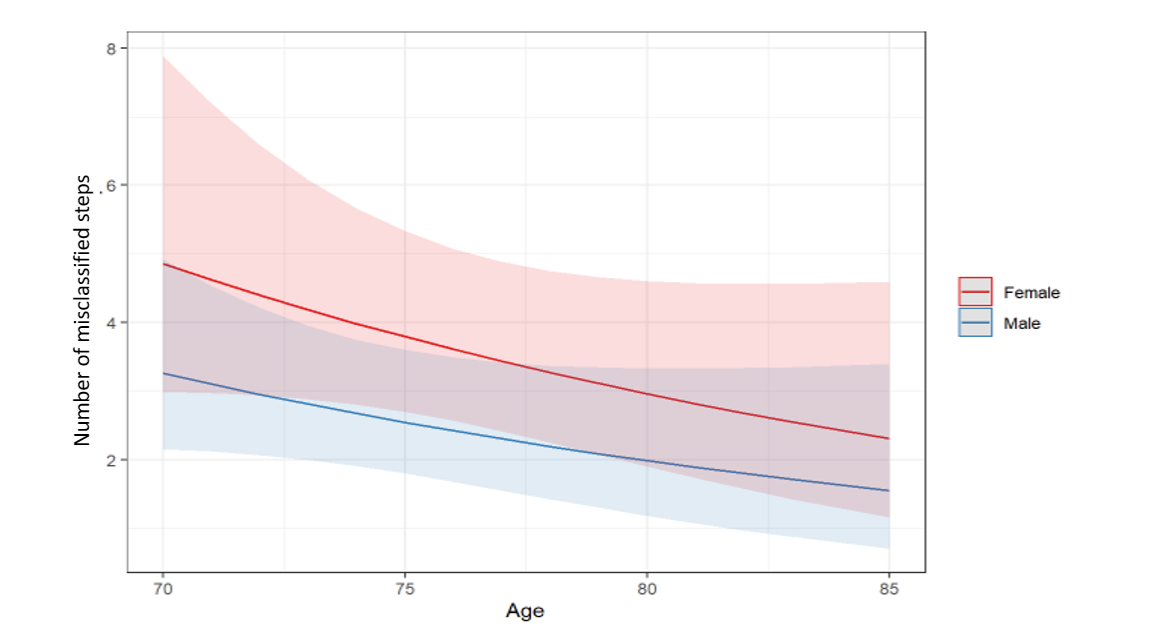
Negative binomial model on the number of wearable device misclassified steps in comparison with the gold standard. The model predictions for the number of misclassified steps, according to sex and age, are reported in the plots. The model estimates, misclassification rate ratio with standard errors SE, and *P* values are reported in the tables. A .94 misclassification rate ratio indicates a 6% reduction in the misclassification rate (over the total number of steps) for a 1-year age increase. The overdispersion estimate is 2.12 (*P*<.001).

**Figure 3:**
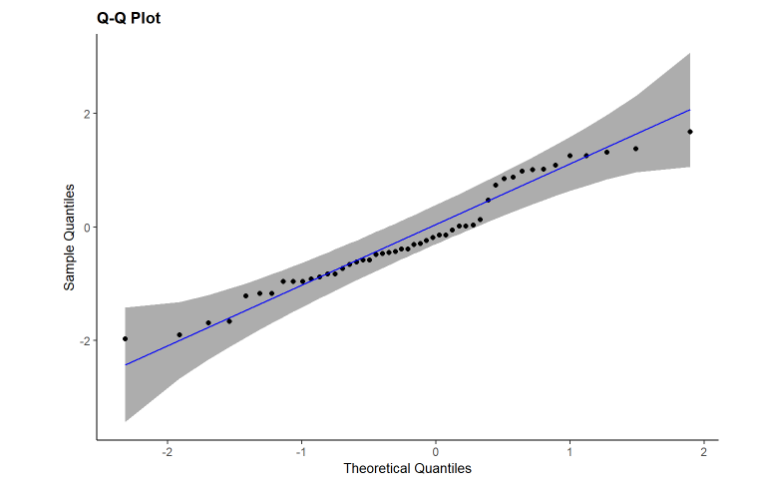
Residual Q-Q plots for the negative binomial models with 95% confidence bounds represented.

The multivariable negative binomial estimate reveals a nonsignificant age and sex effect on the number of misclassified steps (Figure 2). The residuals Q-Q plot indicates a good model fit performance for the negative binomial parametrizations (Figure 3).

## Discussion

### Principal Findings

The purpose of the study was to determine the level of accuracy of a wearable nonmedical device that is widely available in nonspecialized stores in measuring PA in elderly individuals in a real-life setting. Collected data show that a wrist-wearable device is a reliable tool for measuring the level of PA in an elderly population in the context of daily life.

Given its potential and level of accuracy, a wearable device could be used by health care professionals to monitor the levels of PA in their patients. The application of these devices could be easily adopted in situations where there is a need to perform PA for the maintenance or improvement of patient health—for example, following orthopedic or major surgery or to achieve a beneficial increase in metabolism such as in the presence of diabetes or obesity [31-33]. Since these devices are commercially available, inexpensive, specific, and reliable, they could be used in today’s health care environment, where the use of technological tools and adoption of telemedicine methods is becoming increasingly widespread. Wearable devices in health care are seen by older adults as a possible way to improve their health [5]. Indeed, they wish that devices were available in pharmacies, that they could learn about the devices from a health care professional, similar to other health monitoring systems (eg, blood pressure and blood glucose meters), and that health care professionals would use device-collected data.

Activity trackers are not taxed if prescribed in Canada [34], and in 2016 [5], a medical-grade exercise prescription device was recognized as a class 1 medical device. In the United States, a fitness tracker device is eligible for reimbursement when used to treat a medical condition such as obesity [35].

Are future challenges for wearable devices related to reimbursement by health care systems? Current research pipelines aim to collect data to validate their medical relevance to benchmark them against existing clinical solutions, reducing accuracy and reliability issues. Medically relevant clinical data should promote the integration of wearable devices into medical technologies, allowing a rethinking of cost covering by insurance companies and health care systems for clearly defined patient categories. Some examples of wearable systems are starting to be considered eligible for reimbursement [5]. The technology is advancing rapidly, and the market for wearable technology will expand significantly. Despite potential restraints and barriers, such data could cause a dramatic shift in the future of the life and health insurance industry. The evolution of wearable technology in health care is expected to revolutionize the health insurance industry, according to a new report from Timetric’s Insurance Intelligence Center [36].

From a clinical perspective, it may be an important tool for studying the complete 24-hour activity cycle. A wearable device such as the one used in this study could also be easily adopted to measure 24-hour activity in elderly subjects for self-monitoring of spontaneous PA and/or sedentary behavior to prevent weight gain/regain in older adults and for self-monitoring of the effects of PA on self-efficacy and behavior in people with type 2 diabetes. Wearable devices could also allow closer PA monitoring of elderly individuals by both formal and informal caregivers, providing high efficiency for reacting to changes in behaviors.

### Limitations

Study participants were aged 70 to 90 years, with median age being 75 years, a relatively young population considering actual life expectation. Data from this study should be applied with caution in older people. Subjects were selected excluding those needing any walking aid or with any walking disabilities; these study data cannot provide information regarding accuracy in assessing PA in subjects needing those aids or with walking disabilities. Moreover, further research developments are needed for extensive validation in real-life conditions. However, this study could represent a starting point for extending the use of these devices in elderly people, not only for clinical reasons but also for the health monitoring of a population that would benefit greatly from practicing a constant PA.

### Conclusions

Results provided by this study could be considered a good starting point to plan further research considering the collection of different variables and more extensive observations. The device used in this study has the potential to capture the low levels of PA commonly performed by older adults which can be difficult to capture, including activities of daily living, but vital for maintaining health, independence, and quality of life with aging.

### Final Remarks

The level of accuracy of wearable devices in quantifying the PA of older people in a real-life setting that was found in this study supports the idea of considering wrist-wearable nonmedical devices widely available in nonspecialized stores as reliable tools.

## Abbreviations

GLMM: generalized linear mixed-model
GLM: generalized linear model
ICC: intraclass correlation coefficient
LRT: likelihood ratio test
PA: physical activity
